# Electromagnetic Modelling of Fiber Sensors for Low-Cost and High Sensitivity Temperature Monitoring

**DOI:** 10.3390/s151229770

**Published:** 2015-11-30

**Authors:** William Scarcia, Giuseppe Palma, Mario Christian Falconi, Francesco de Leonardis, Vittorio M. N. Passaro, Francesco Prudenzano

**Affiliations:** Department of Electrical and Information Engineering, Politecnico di Bari, via E. Orabona n. 4, Bari 70125, Italy; w.scarcia@live.it (W.S.); giuseppe.palma@poliba.it (G.P.); christian.falconi@poliba.it (M.C.F.); francesco.deleonardis@poliba.it (F.D.L.); vittorio.passaro@poliba.it (V.M.N.P.)

**Keywords:** optical fiber, temperature optical sensor, electromagnetic modeling, electromagnetic noise immunity

## Abstract

An accurate design of an innovative fiber optic temperature sensor is developed. The sensor is based on a cascade of three microstructured optical fibers (MOFs). In the first one a suitable cascade of long period gratings is designed into the core. A single mode intermediate and a rare-earth activated Fabry-Perot optical cavity are the other two sensor MOF sections. An exhaustive theoretic feasibility investigation is performed employing computer code. The complete set-up for temperature monitoring can be obtained by utilizing only a low cost pump diode laser at 980 nm wavelength and a commercial optical power detector. The simulated sensitivity *S* = 315.1 μW/°C and the operation range ΔT = 100 °C is good enough for actual applications.

## 1. Introduction

During the last decade, several innovative temperature optical sensors have been proposed in the literature. A number of different strategies have been successfully applied in order to obtain feasible and reliable temperature sensing, e.g., by exploiting the temperature dependence of integrated systems and/or single elements based on fiber Bragg gratings (FBGs), long period gratings (LPGs), Fabry-Perot (FP) cavity lasers, Sagnac loops, interferometers, off-set spliced fibers, *etc*. [1,2,3,4,5,6,7,8,9,10,11,12,13,14,15,16,17,18,19]. Intrinsic FP fiber-optic temperature sensors can be obtained by employing the variation with temperature of the reflectivity exhibited by a fiber splice. In particular, different core diameter fibers can be joined to obtain the optical beam reflection; the temperature measurement can be performed by measuring the spectral fringe changes [1]. Two core-offset joins act as an interferometer and the temperature applied to the core-offset structure induces a proportional wavelength shift. A single mode fiber (SMF) core-offset structure can be put within a ring laser. The central wavelength shift of the ring cavity laser including the core-offset structure is proportional to the temperature change. By following this strategy, a sensitivity of 0.0449 nm/°C in the range of 30–270 °C was demonstrated [2]. In [3] the FP cavity was formed by splicing a side-hole microstructured fiber (MF) and a solid SMF on a fuse-silica tube; a high gas pressure was obtained inside the FP cavity through the MF holes. In [4] a liquid-filled MOF Mach–Zehnder interferometer was proposed. Fiber optic reels can be optimized to obtain temperature sensors based on a Sagnac loop [5]. Conventional technologies can also be successfully exploited to construct high performance sensors. As an example, single mode multimode-single mode (SMS) fiber structures can be constructed by cleaving and splicing processes [6,7]. In reference [7] a temperature sensor is constructed by fixing a bent SMS fiber on a polymer plate frame. The sensor operation is based on the curvature change of the bent SMS fiber due to the swelling/shrinking of the polymer plate frame caused by the temperature change. The central wavelength shift of the sensor output spectrum allows the measurement of the temperature, which can be determined with a maximum sensitivity of 6.5 nm/°C in the range from 51 °C to 65 °C. A high temperature probe sensor based on a Michelson interferometer was demonstrated in [12], whereby a sensitivity of 0.140 nm/°C from 30 °C to 800 °C was achieved, and the linearity was 99.9%.

Unfortunately, in most of the cases reported in literature, optical fiber sensor systems are not fully integrated and directly usable. They are refined laboratory prototypes but, generally, they do not include an integrated electronics for detection and/or signal-processing. They require further engineering to be launched on the market. More precisely, most of these sensors require the addition of sophisticated and high cost set-ups to perform complete temperature measurement. As an example, the evaluation of the central wavelength shift of FP, FBG, LPG sensors, in most cases, implies the use of optical spectrum analyzers (OSAs) or *ad hoc* optical circuitry, including optical to electrical conversion and/or interferometric sections.

In this paper, an accurate theoretical investigation on the feasibility of an innovative fiber optic temperature sensor, conceived as a standalone device, is performed. It is based on a cascade of three integrated/spliced microstructured optical fibers (MOFs). In the first one a suitable cascade of LPGs is designed into the core. A single mode intermediate MOF and an ytterbium doped MOF laser are the other two sensor sections. The three MOFs are designed to be easily spliced. The complete design is performed by employing an ad hoc computer code developed for this purpose. Numerous simulations have been performed to refine the sensor behavior. The strong interest in this kind of device is that, in spite of its design complexity, it can be fabricated via conventional techniques. Moreover, a complete set-up for temperature detection could be obtained by utilizing, in addition to the proposed sensor, only a low cost pump diode laser at 980 nm wavelength and a commercial optical power meter. This kind of optical sensor exhibits all the well-known fiber optic properties, e.g., immunity to electromagnetic noise and compactness, furthermore it can be optimized to monitor different temperature ranges via a proper choice of the gratings. The paper illustrates the sensor characteristics, theoretically evaluated through a number of accurate simulations. This analysis can be considered a feasibility investigation, realistically performed before the final device fabrication.

The paper is organized as follows: in Section 1 the Introduction is presented, followed in Section 2 by a brief description of sensor operation and the corresponding theory. Section 3 presents the strategy for sensor design and optimization, and in Section 4 the conclusions are given.

## 2. Sensor Operation and Theory

### 2.1. Sensor Operation

**Figure 1:**
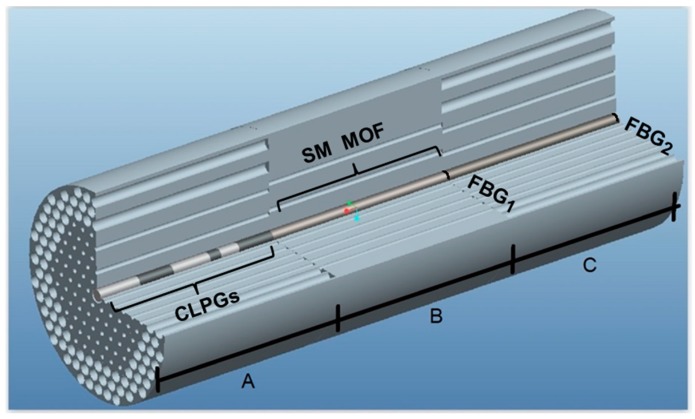
Sections of the temperature sensor: Section A: double cladding MOF with CLPGs inscribed within the core; Section B: single mode intermediate MOF; Section C: ytterbium-doped MOF laser cavity.

In this paragraph, before describing the model, the sensor operation is briefly illustrated. Figure 1 illustrates the sensor. Its structure is based on a cascade of three photonic crystal fibers. In the first one a suitable cascade of long period gratings (CLPGs) is designed into the core. A single mode (SM) intermediate fiber and an ytterbium doped fiber laser are the other two sensor sections. The three photonic crystal fibers are designed to be spliced by maintaining the optical field matching. More precisely, they have the same outer cladding diameter, the same core diameter and the same refractive index change between the core and the cladding.

**Figure 2:**
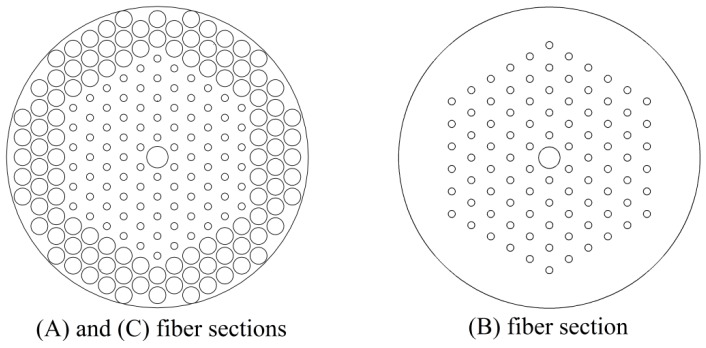
Transversal geometry of the three sensor sections: (**A**) Double cladding MOF with CLPGs inscribed within the core; (**B**) Single mode intermediate MOF; (**C**) Ytterbium-doped MOF laser cavity.

The Section A sensor (see Figure 1 and Figure 2), is a microstructured optical fiber (MOF) with a multimodal inner cladding. Some cladding modes at the pump wavelength λ_p_ are coupled with the fundamental core mode at the pump wavelength through the CLPGs. In the CLPGs each LPG has a different length and period in order to selectively couple peculiar cladding modes with the fundamental core mode at the pump wavelength λ_p_. The power of the fundamental core mode at the pump wavelength λ_p_ is increased since some cladding modes, coupled by the CLPGs, transfer their power towards the core. The power of the fundamental core mode, at the end of the Section A sensor, depends on the temperature. This is due to the temperature dispersion of the MOF refractive index and to the glass thermal expansion which slightly changes the sensor geometry, *i.e.*, the fiber section, the length and the period of the gratings. However, the aforesaid dependence is too slight to be directly exploited for a sensor.

The Section B sensor (see Figure 1 and Figure 2), is a single mode intermediate MOF. The cladding modes coming from the Section A sensor are not guided in the Section B sensor due to the lack of the larger external holes. The Section B sensor prevents further exchange of power at the pump wavelength λ_p_ from the core towards the cladding, since only the core mode is guided. In particular, the Section B sensor should be longer than a few wavelengths to attenuate almost completely the residual power of the cladding modes and long enough to be spliced with both Section A and Section C. Other techniques as tapering, or cladding removal could be employed to obtain a similar effect.

The Section C sensor is an *ad hoc* Fabry-Perot, rare-earth activated, optical cavity. More precisely, it is an ytterbium doped laser constituted by a MOF having a cross section equal to that of the Section A sensor. The optical cavity is obtained by employing two fiber Bragg gratings, FBG_1_ and FBG_2_ and the core is ytterbium doped. The laser is suitably designed in order to obtain a signal power proportional to the temperature variation with a small zero-offset value. More precisely, it works close to the threshold pump power. The signal laser intensity depends on the core fundamental mode power at pump wavelength, coming from the Section B sensor end.

### 2.2. Theory

The modal electromagnetic field profile and the propagation constant of the guided modes are calculated via a full vectorial Finite Element Method (FEM) based solver. For the wavelength dispersion of silica a suitable Sellmeier equation is employed [20].

For the Section A sensor, the core refractive index perturbation *Δn*(*z*) along the MOF longitudinal direction *z*, constituting the CLPGs, can be easily fabricated by employing conventional technology [21,22,23,24]. The refractive index change is uniform in the fiber transverse plane and it is expressed as in [25,26]:

(1) $$\Delta n\left(z\right)=n_{1}\psi \left(z\right)\left[1+m\cos\left(2\pi z/\Lambda \right)\right]$$

where *n_1_* is the unperturbed core refractive index, Λ is the grating period of each grating constituting the CLPGs, *m* is the induced-index fringe modulation, and ψ(z) is the slowly varying envelope of the grating.

The thermal sensitivity of the CLPGs is modeled by considering the change of refractive index and the thermal expansion of silica *versus* the temperature *T* [27]:

(2) $$\Delta n\left(z,T\right)=n_{1}\psi \left(z\right)F\left(z\right)+{\frac{\partial n}{\partial T}|}_{T=T_{E}}\left(T-T_{E}\right)+\left[n_{1}+n_{1}\psi \left(z\right)F\left(z\right)-1\right]\left(1+\rho \right)\alpha_{T}\left(T-T_{E}\right)$$

where *T_E_* is the environment temperature, F(z)=[1+mcos(2πz/Λ)], *ρ* = 0.16 is the Poisson’s ratio of silica, $\alpha_{T}=0.51\times 10^{-6}\;K^{-1}$ is the thermal expansion coefficient of silica, ${\frac{\partial n}{\partial T}|}_{T=T_{E}}=10^{-5}\;K^{-1}$ is the derivative of refractive index with respect to the temperature [27].

The thermal expansion of the glass is taken into account by employing the following formula:

(3) $$L=L_{0}\left[1+\alpha_{T}\left(T-T_{E}\right)\right]$$

where *L* is the length of the glass segment at the temperature *T* and *L_0_* is the reference length at the environment temperature *T_E_*.

The coupled mode equations, describing the electromagnetic field propagation along the CLPGs inscribed in the Section A sensor, are written by considering the Slowly Varying Envelope Approximation (SVEA), *i.e.*, for ψ(z)<<1 [25]. The coupled equation system integration allows the calculation of the amplitude variation of the transverse field profiles of the forward propagation modes at the pump wavelength along the propagation direction, via the transverse coupling coefficients between the interacting modes [25,26]. The longitudinal coupling coefficients can be neglected because they are about three orders of magnitude smaller than the transverse coupling coefficients [25].

The electromagnetic field profile of the ν-th mode at the wavelength λ is normalized to unity:

(4) $$\iint_{S}{Ε}_{\nu }(x,y)\cdot {Ε}_{\nu }^{*}(x,y)dxdy=1$$

where *S* is the whole MOF cross section including both the core and the cladding regions and ${Ε}_{\nu }(x,y)$ is the electric field profile of the *ν*-th mode into the fiber. The coupled mode equations for *N* interacting modes are:

(5) $$\frac{dA_{\mu }^{P}}{dz}=i\sum_{\nu =1}^{N}A_{\nu }^{P}K_{\nu \mu }\exp\left[i\left(\beta_{\nu }-\beta_{\mu }\right)z\right]\;\mu =1,2,...,N$$

where $A_{\mu }^{P}$ and $A_{\nu }^{P}$ are the amplitudes of the μ-th and ν-th modes at the pump wavelength, $\beta_{\mu }$ and $\beta_{\nu }$ are the propagation constants of the modes *μ* and ν, $K_{\nu \mu }$ is the transverse coupling coefficient between modes ν and *μ*, it is expressed by Equations (6) and (7):

(6) $$K_{\nu \mu }(z)=k_{\nu \mu }(z)\left[1+m\cos\left(2\pi z/\Lambda \;\right)\right]$$

(7) $$k_{\nu \mu }(z)=\frac{\omega \varepsilon_{o}n_{1}^{2}\psi (z)}{2}\iint_{S_{c}}{Ε}_{\nu }(x,y)\cdot {Ε}_{\mu }^{*}(x,y)dxdy$$

where ω is the radian frequency. The surface integral is calculated over the fiber core section *S_c_*, where the grating is written. The strength of the interaction, *i.e.*, power exchange, about the coupled modes depends (i) on the grating period Λ, properly chosen to compensate the phase mismatch, $\beta_{\nu }$ − $\beta_{\mu }$ between the modes ν and *μ* and (ii) $k_{\nu \mu }$ *i.e.*, the overlapping between the electromagnetic field profiles ${Ε}_{\nu }$ and ${Ε}_{\mu }$ of the modes ν and *μ*.

An input pump power uniformly distributed among all the propagating cladding modes is considered in the simulation. In fact, in actual cases, due to the fabrication tolerances, the inner cladding exhibits small random physical changes (in size, shape, refractive index profile). As a consequence, the cladding modes are coupled to each other in such a way that, after the propagation along a number of wavelengths, each mode exhibits almost the same power [28].

For the Section C sensor, the quasi-two level scheme is employed in order to model the ytterbium activated glass-system. The expression of the ν-th mode power is:

(8) $$\frac{dP_{\nu }\left(z,\lambda_{i}\right)}{dz}=P_{\nu }\left(z,\lambda_{i}\right)\left[\sigma_{21}\left(\lambda_{i}\right)N_{2}\left(z\right)-\sigma_{12}\left(\lambda_{i}\right)N_{1}\left(z\right)\right]\Gamma_{\nu }\left(\lambda_{i}\right)-\gamma \left(\lambda_{i}\right)P_{\nu }\left(z,\lambda_{i}\right)\;i=S,P$$

where *S*, *P* refer to the signal and the pump, respectively; *σ_21_* is the emission and *σ_12_* the absorption ytterbium cross-section; *N_1_* is the population of the ytterbium ions at the ground state ^2^F_7/2_ and *N_2_* the population at the upper laser energy level ^2^F_5/2_; $\gamma \left(\lambda_{i}\right)$ are fiber propagation losses as a function of the wavelength; $\Gamma_{\nu }\left(\lambda_{i}\right)$ is the overlapping coefficient.

The steady state ion populations are:

(9) $$N_{2}=\frac{W_{P}^{GSA}+W_{S}^{GSA}}{W_{P}^{GSA}+W_{S}^{GSA}+1/\tau_{21}+W_{P}^{E}+W_{S}^{E}}N_{Yb}$$

(10) $$N_{1}=N_{Yb}-N_{2}$$

where $W_{P}^{GSA}$ and $W_{S}^{GSA}$ are the ground state absorption (GSA) rates at pump and signal wavelength, respectively; *N_Yb_* is the ytterbium ion concentration; $W_{P}^{E}$ and $W_{S}^{E}$ are the emission (*E*) rates at pump and signal wavelength, respectively; 1/τ21 is the spontaneous emission rate from ^2^F_5/2_ energy level to the ^2^F_7/2_ one; [20,29,30].

The emission and absorption rates at the frequency of the pump *f_P_* and signal *f_S_* are calculated as follows:

(11) $$\begin{array}{l} W_{P}^{GSA}=\sigma_{12}(\lambda_{P})\frac{1}{S_{c}}\frac{P_{\nu }^{P}(z,\lambda_{P})\Gamma_{\nu }\left(\lambda_{P}\right)}{hf_{P}}\\ W_{S}^{GSA}=\sigma_{12}(\lambda_{S})\frac{1}{S_{c}}\frac{P_{\nu }^{S}(z,\lambda_{S})\Gamma_{\nu }\left(\lambda_{S}\right)}{hf_{S}}\\ W_{P}^{E}=\sigma_{21}(\lambda_{P})\frac{1}{S_{c}}\frac{P_{\nu }^{P}(z,\lambda_{P})\Gamma_{\nu }\left(\lambda_{P}\right)}{hf_{P}}\\ W_{S}^{E}=\sigma_{21}(\lambda_{S})\frac{1}{S_{c}}\frac{P_{\nu }^{S}(z,\lambda_{S})\Gamma_{\nu }\left(\lambda_{S}\right)}{hf_{S}} \end{array}$$

where *h* is the Planck constant. Mode optical amplification can occur when the normalized overlapping coefficient $\Gamma_{\nu }$ is large enough, its calculation for the fundamental core mode at the signal wavelength λ_S_ (ν=1) and for the ν-th propagating mode at the pump wavelength λ_P_ (ν≥1), is obtained via:

(12) $$\Gamma_{\nu }\left(\lambda_{i}\right)=\frac{\iint_{S_{c}}{Ε}_{\nu }\left(x,y,\lambda_{i}\right)\times {H_{\nu }}^{*}\left(x,y,\lambda_{i}\right)\cdot u_{z}\;dxdy}{\iint_{S}{Ε}_{\nu }\left(x,y,\lambda_{i}\right)\times {H_{\nu }}^{*}\left(x,y,\lambda_{i}\right)\cdot u_{z}\;dxdy}\;i=S,P$$

*S_c_* is the rare earth doped region, *i.e.*, the core region, **u**_*z*_ is the versor of the z axes, **Ε**_*ν*_ and **H**_*ν*_ are the electric and magnetic field profiles of the electromagnetic mode ν-th. This model is based on a well validated formalism [25,26,27,28,29,30].

## 3. Sensor Design and Optimization

The main optical and geometrical MOF sensor parameters employed in the simulation are summarized in Table 1, Table 2 and Table 3, which refer to Section A, Section B and Section C of the sensor, respectively, as illustrated in Figure 1 and Figure 2.

The parameters pertaining to the fiber transversal geometries are identified in order: (i) to maximize the core mode area (*i.e.*, to obtain a large mode area fiber), by maintaining the single mode propagation in the core at both the pump and the signal wavelengths; (ii) to reduce power density within the multimode inner cladding, at the pump wavelength. Therefore, low power density is confined within the MOF. This avoids undesired thermal effects which could be directly dependent on the optical power propagation rather than on the external temperature.

The pump and signal wavelengths are *λ_P_* = 976 nm and *λ_S_* = 1060 nm, respectively. In the three fiber sections all the circles represent air holes, with the exception of the central one which is a higher refractive index core. The core refractive index *n_c_* and the cladding refractive index *n_clad_* at the pump and signal wavelengths are *n_c_*(*λ_P_*) = 1.45172, *n_c_*(*λ_S_*) = 1.45067, *n_clad_*(*λ_P_*) = 1.45072, *n_clad_*(*λ_S_*) = 1.44967, according to the Sellmeier equation [20].

**Table 1:** Section A sensor.

	Laser Parameter	Value
*D_out_*	Outer fiber diameter	107 μm
*d_c_*	Core diameter	7.6 μm
Λ_p_	Hole pitch	7 μm
*d_h_*	Diameter of inner cladding holes	2.5 μm
*D_h_*	Diameter of outer cladding holes	6 μm

**Table 2:** Section B sensor.

	Laser Parameter	Value
*D_out_*	Outer fiber diameter	107 μm
*d_c_*	Core diameter	7.6 μm
Λ_p_	Hole pitch	8 μm
*d_h_*	Diameter of inner cladding holes	2.5 μm

**Table 3:** Section C sensor.

	Laser Parameter	Value
*D_out_*	Outer fiber diameter	107 μm
*d_c_*	Core diameter	7.6 μm
Λ_p_	Hole pitch	7 μm
*d_h_*	Diameter of inner cladding holes	2.5 μm
*D_h_*	Diameter of outer cladding holes	6 μm
*τ_21_*	Ytterbium laser lifetime	0.8 ms
*σ_12_*(*λ_p_*)	Absorption cross sections at pump wavelength	2.6272 × 10^−24^ m^2^
*σ_21_*(*λ_signal_*)	Absorption cross sections at signal wavelength	6.1477 × 10^−27^ m^2^
*σ_12_*(*λ_p_*)	Emission cross sections at pump wavelength	2.5617 × 10^−24^ m^2^
*σ_12_*(*λ_signal_*)	Emission cross sections at signal wavelength	3.1774 × 10^−25^ m^2^
*γ*(*λ_P_*)	Fiber losses at pump wavelength	4.2 dB/Km
*γ*(*λ_S_*)	Fiber losses at signal wavelength	2 dB/Km
*N_Yb_*	Ytterbium ion concentration	5 × 10^25^ ions/m^3^
*R_1_*	Input mirror reflectivity	0.99
*R_2_*	Output mirror reflectivity	0.06

A slight refractive index change *Δn* = 0.001 between core and cladding, in the Section C sensor, is due to the ytterbium ions. In the Section A sensor and Section B sensor the same refractive index change is obtained by doping the core with germanium, to avoid reflections along the propagation direction.

The CLPGs of the Section A sensor are modeled via Equation (1) for an induced-index fringe modulation *m* = 1, and $n_{1}\psi (z)=\text{cost}=1\times 10^{-4}$. Therefore $k_{\nu \mu }$ is constant with respect to *z* propagation direction.

The FEM investigation is performed for calculating the electromagnetic field profile and the propagation constant of the fundamental core modes ($HE_{11}^{x}$ and $HE_{11}^{y}$). About four hundred inner cladding modes are calculated. Among these cladding modes, those effectively involved in the energy transfer towards the fundamental core mode are only 64. These modes have stronger coupling coefficient $K_{\nu \mu }$ and stronger overlapping coefficient $\Gamma_{\nu }$ than those pertaining to the other ones. By considering the mode degeneracy, the two fundamental core modes are identified with the M_1_ solution and the 64 degenerated cladding modes are identified with the names M_2_, M_3_, … M_33_. More precisely, a system of 33 coupled equations (Equation (5) for *N* = 33) is integrated to calculate the evolution of the mode amplitudes along the Section A sensor at the pump wavelength.

Initially, the period and length of each LPG of the Section A sensor, *i.e.*, CLPGs, are designed for increasing the core power at the pump wavelength. The input pump power *P_P_* = 1 W, launched in the Section A sensor, is considered in the simulations if not differently specified.

**Figure 3:**
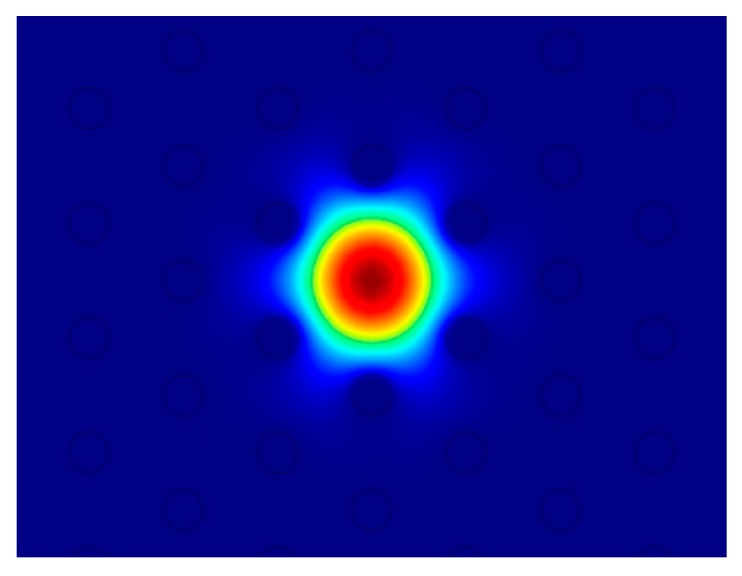
Norm of the electromagnetic field distribution of the M_1_ mode guided in the core at the pump wavelength.

**Figure 4:**
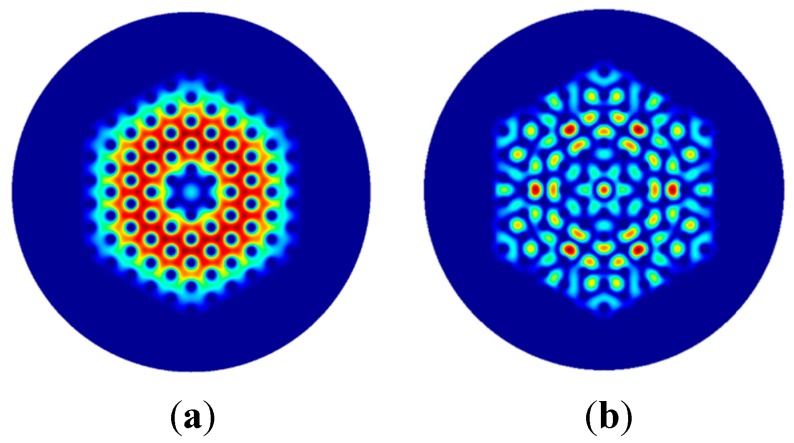
Norm of the electromagnetic field distribution at the pump wavelength of: (**a**) M_4_ cladding mode; (**b**) M_21_ cladding mode.

The phase matching among selected cladding modes and the fundamental core mode allows the power transfer from the cladding towards the core to be achieved [25,26]. This condition is reached via gratings with peculiar values of the period Λ. The competition among the interacting modes is considered by writing the coupled equation system, Equation (5), for different grating periods Λ, via a parametric investigation. Arbitrary examples of cladding modes which can exchange power with the fundamental core mode at pump wavelength are M_4_ and M_21_. M_4_ is the lower order cladding mode which interacts with the fundamental one. It exhibits an electromagnetic field profile which overlaps the core region (*i.e.*, the electromagnetic field profile of M_1_ mode). Therefore, a not negligible coupling coefficient $k_{14}$ = 18 × 10^−4^ is calculated. A similar comment can be made for M_21_, for which the coupling coefficient is $k_{1\text{21}}$ = 1.78 × 10^−2^. Figure 3 illustrates the norm of the electromagnetic field distribution of the M_1_ mode guided in the core at the pump wavelength. Figure 4a shows the electromagnetic field distribution of the M_4_ cladding mode. Figure 4b refers to the electromagnetic field distribution of the M_21_ cladding mode. The blue color indicates lower electromagnetic field intensity while the red color refers to higher intensity. These figures are not in the same scale to increase their readability. They qualitatively demonstrate that the integral of Equation (7) calculating the overlapping of M_1_ with M_4_ or M_21_ is not negligible in both cases.

**Figure 5:**
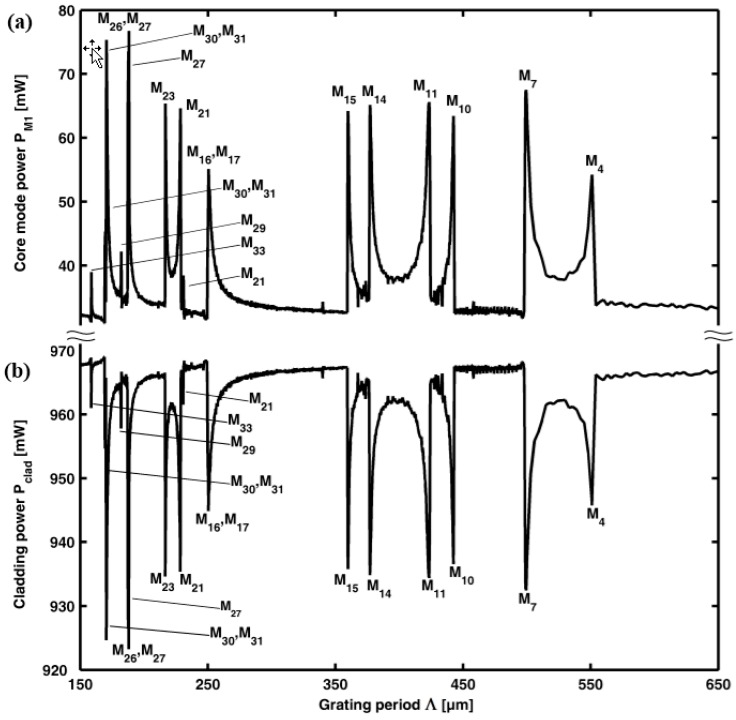
Resonances for a LPG inscribed into the MOF of the Section A-sensor *versus* the grating period Λ, at the pump wavelength *λ_P_*, LPG length L = 10 cm: (**a**) Core mode power P_M1_(*λ_P_*); and (**b**) Cladding power *P_clad_*.

Indicative values of the grating periods allowing the mode interaction can be roughly evaluated by the inspection of Figure 5. Figure 5a illustrates, the core mode power P_M1_ of the fundamental mode M_1_ (HE_11_), at the pump wavelength *λ_P_*, as a function of the grating period Λ, for an arbitrary grating length *L* = 10 cm. Figure 5b gives complementary information. It illustrates the total power guided by the inner cladding, *P_clad_*, *i.e.*, the sum of all the interacting cladding mode M_2_-M_33_ powers.

The peaks of the core mode power P_M1_ in Figure 5a such as the deeps of the cladding power *P_clad_* of Figure 5b correspond to the particular grating periods Λ_R_ which enable the power exchange from the inner cladding modes M_2_-M_33_ towards (troughs) the fundamental M_1_ mode (peaks) at the pump wavelength, since they allow the phase matching. The calculation is performed at the temperature *T* = 25 °C. Sixteen different grating periods enable the power exchange. For each single grating period *Λ_R_*, the length L_Gmaxc_ providing the maximum power exchange from the cladding modes M_2_-M_33_ towards the fundamental M_1_ can be identified through a parametric simulation. The obtained results are useful to understand the behavior of each grating optimized as a standalone device, *i.e.*, not included within the cascade of gratings.

Table 4 illustrates the grating periods *Λ_R_*, the peculiar cladding mode M_i_ coupled to the fundamental M_1_ one, the grating length L_Gmaxc_, the percentage increase of the M_1_ power at the end of grating L_Gmaxc_.

In order to obtain a temperature sensor, a cascade of gratings CLPGs with different periods and lengths is designed. The first goal is to obtain a variation of the fundamental mode M_1_ power *versus* the temperature, at the end of CLPGs larger than that obtainable with only one of the singularly-optimized gratings reported in Table 4.

Negative/positive variations of temperature with respect to the environment value induce an effect similar to a slight decrease/increase in fiber length, grating period, refractive index according to Equations (2) and (3). As a consequence, the coefficients of the 33 coupled equation system, Equation (5), depend on temperature. To obtain a linear and univocal sensor response *versus* the temperature, e.g., in the range from 0 °C to 100 °C, different grating periods must be accurately identified. Starting from the values reported in Table 4 all the gratings of the cascade are optimized in order to identify: (i) the period *Λ_opt_* causing a decreasing of the M_1_ power when the temperature changes from 0 °C to 100 °C; (ii) the length allowing the largest variation of the M_1_ power, at the their end, for the same temperature change.

**Table 4:** Gratings maximizing the power exchange from the inner cladding modes M_2_-M_33_ towards the fundamental core mode M_1_ at the environmental temperature 25 °C.

Grating	Λ_R_ (μm)	Coupled Cladding Modes	L_Gmaxc_ (cm)	P_M1maxc_ (mW)	Percent Increase of M_1_ Power
R_1_	551.0	M_4_	5.400	54.23	78.96%
R_2_	499.0	M_7_	7.885	67.3	122.09%
R_3_	442.4	M_10_	9.470	63.42	109.29%
R_4_	423.4	M_11_	6.270	65.59	116.45%
R_5_	377.0	M_14_	7.980	65.15	115.00%
R_6_	359.8	M_15_	9.080	64.19	111.83%
R_7_	250.4	M_16.M17_	9.445	54.84	81.86%
R_8_	230.6	M_21_	8.650	38.44	26.85%
R_9_	228.4	M_21_	4.705	64.61	113.21%
R_10_	216.6	M_23_	7.98	65.27	115.72%
R_11_	188.0	M_26.M27_	8.480	76.75	153.28%
R_12_	187.6	M_27_	2.655	73.53	142.65%
R_13_	182.0	M_29_	7.435	42.19	39.23%
R_14_	170.4	M_30_,M_31_	3.100	75.31	148.52%
R_15_	169.4	M_30_,M_31_	7.140	45.31	49.52%
R_16_	158.6	M_33_	9.38	38.98	28.63%

As an example, by starting from reference the value *Λ_R_* = 228.4 μm, reported in Table 4 for the R_9_ grating, an optimal nominal value *Λ_opt_* = 228.8 μm is chosen. In fact, this value allows an almost linear and decreasing variation of the M_1_ core mode power P_M1_ for an increasing of the grating period from 228.4 μm to 229.2 μm. This criterion is qualitatively adopted since the increase of temperature causes an increase of grating period and length. By following this procedure, at first, sixteen different tentative gratings allowing a decreasing M1 power by increasing the period (temperature) were identified.

Among these sixteen gratings, only a part of them allows a large energy transfer (ΔP_M1_ > 1.5 mW) from the cladding mode towards M_1_. Moreover, some gratings are neglected because they are redundant ones (as example, when their grating periods are too close to other ones previously employed in the cascade). Therefore, only seven gratings are considered for the first tentative of cascade CLPG_1_. Table 5 reports the parameters of the seven gratings with the period *Λ_opt_* which allows a decreasing M_1_ power ΔP_M1_ by increasing the temperature, the length allowing the maximum variation of ΔP_M1_ and the corresponding ΔP_M1_ (negative value indicates a decrease). Also in this case each grating length L_ΔPM1max_ is optimized separately from the other gratings in order to obtain the maximum variation of ΔP_M1_ when the temperature varies from 0 °C to 100 °C.

**Figure 6:**
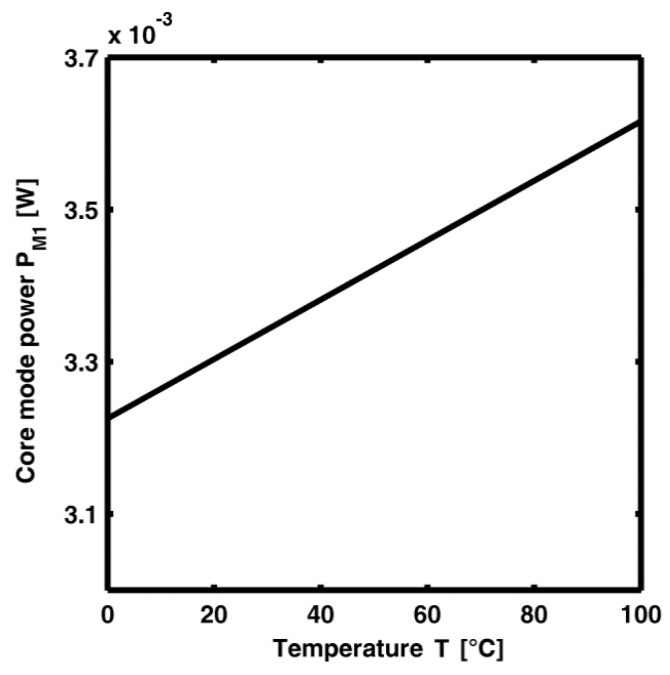
Power of the fundamental core mode M_1_ at the pump wavelength *versus* the temperature, at the output section of CLPG_1_ when each LPG is optimized separately.

Figure 6 illustrates the power of M_1_ mode *versus* the temperature at the end of CLPG_1_, *i.e.*, after the cascade of the seven gratings listed in Table 5. The maximum change ΔP_M1_ = 0.39 mW is very slight. The obtained M_1_ power change is positive (instead of negative) for a variation of temperature from 0 °C to 100 °C.

**Table 5:** Parameters of grating cascade CLPG_1_, grating length L_ΔPM1max_ separately optimized.

Grating	Λ_opt_ (μm)	L_ΔPM1max_ (cm)	ΔP_M1_ (mW)
$R_{1}^{CLPG1}$	188.2	8.09	−3.752
$R_{2}^{CLPG1}$	169.5	7.14	−2.642
$R_{3}^{CLPG1}$	228.8	7.15	−2.004
$R_{4}^{CLPG1}$	182.2	9.68	−1.786
$R_{5}^{CLPG1}$	216.8	7.98	−1.674
$R_{6}^{CLPG1}$	250.6	9.45	−1.608
$R_{7}^{CLPG1}$	443.2	9.43	−1.554

This result is not useful and it is reported only for a comparison. Since each grating of CLPG_1_ is optimized independently from the other ones, their single contribution to the M_1_ power change, ΔP_M1_, can be opposite to that of other gratings when they are in cascade. Therefore, the overall CLPG_1_ behavior is not efficient for temperature sensing. More precisely, the length of each LPG of CLPG_1_ induces a phase shift of the M_1_ mode which can give an undesired effect (mitigating the temperature dependence).

**Figure 7:**
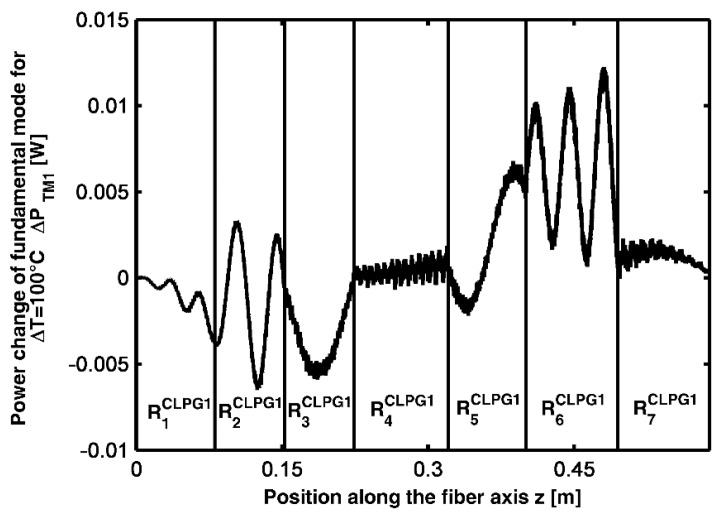
Power change of the M_1_ mode, ΔP_M1ΔT_, for the temperature variation 0–100 °C, along the grating cascade CLPG_1_.

**Figure 8:**
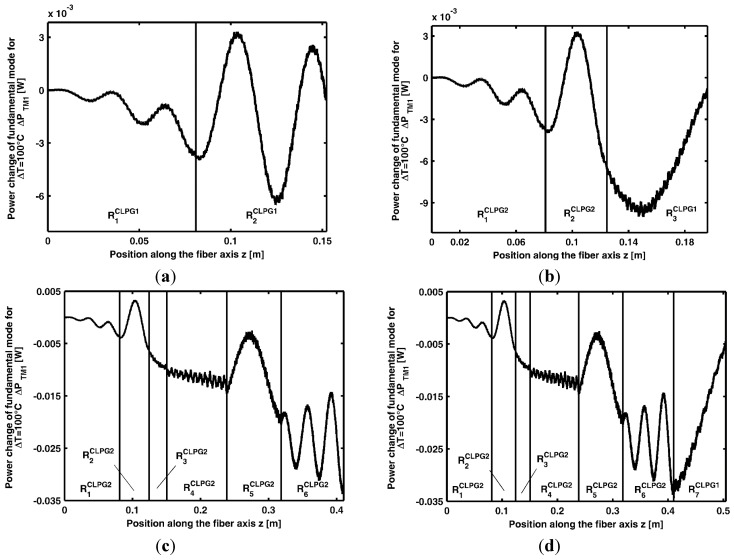
Power change of the M_1_ mode, ΔP_M1ΔT_, for the temperature variation 0–100 °C (**a**) along the grating cascade made of the first two gratings of CLPG_1_; (**b**) along the grating cascade made of the first two optimized gratings of CLPG_2_ and the third grating of CLPG_1_; (**c**) along the grating cascade CLPG_2_; (**d**) along the cascade made of the six gratings of Table 6 and the seventh grating of CLPG_1_.

After this first attempt, the cascade CLPG_2_ is globally optimized. To perform the global optimization, in the simulation of the cascade CLPG_2_, the optimal grating lengths are identified by considering as input of each single grating the output of the previous one. This design procedure is more correct. In this last case, six gratings allow to obtain the maximum output power variation, for a variation of temperature from 0 °C to 100 °C. The parameters of CLPG_2_, parametrically investigated, are listed in Table 6. For a comparison between CLPG_1_ and CLPG_2_, and in order to illustrate the design strategy Figure 7 and Figure 8a–d are shown.

**Table 6:** Parameters of grating cascade CLPG_2_, grating length L_ΔPM1max_ globally maximizing the decreasing of M_1_ power, ΔP_M1,_ when the temperature varies from 0 °C to 100 °C.

	Λ_opt_ (μm)	L_Δ__PM1max_ (cm)	Total ΔP_M1_ (mW)
$R_{1}^{CLPG2}$	188.2	8.09	−33.46
$R_{2}^{CLPG2}$	169.5	4.36	
$R_{3}^{CLPG2}$	228.8	2.60	
$R_{4}^{CLPG2}$	182.2	8.79	
$R_{5}^{CLPG2}$	216.8	7.98	
$R_{6}^{CLPG2}$	250.6	9.20	

Figure 7, pertaining to CLPG_1_, illustrates the power change of the M_1_ mode, ΔP_M1ΔT_, along the cascade of the seven gratings listed in Table 5, separately optimized, for a variation of temperature from 0 °C to 100 °C. The vertical lines indicate the different gratings. Since each grating of cascade CLPG_1_ is optimized independently from the others, ΔP_M1ΔT_ is slightly increased at the end of CLPG_1_.

Figure 8a depicts the power change of the M_1_ mode, ΔP_M1ΔT_, for the temperature variation 0–100 °C, along the grating cascade made of the first two gratings of CLPG_1_, see Table 5. The maximum power change of the M_1_ mode, ΔP_M1ΔT_, along the cascade of the two gratings is obtained for the $R_{1}^{CLPG2}$ length L_ΔPM1max_ = 8.09 cm and for th $R_{2}^{CLPG2}$ length L_ΔPM1max_ = 4.36 cm, see Table 6. In other words, starting from CLPG_1_ gratings after this first optimization, $R_{1}^{CLPG2}$ and $R_{2}^{CLPG2}$ of CLPG_2_ are identified as in Table 6.

Figure 8b depicts the power change of the M_1_ mode, ΔP_M1ΔT_, for the temperature variation 0–100 °C, along the grating cascade made of the first two optimized gratings, of CLPG_2_, and the third grating of CLPG_1_. The maximum power change of the M_1_ mode, ΔP_M1ΔT_, along the cascade of the three gratings is obtained by considering a third grating having length L_Δ__PM1max_ = 2.60 cm. This is the third optimized length L_Δ__PM1max_ reported in Table 6. The described design procedure allows an increasing of the sensor performance till the addition of the sixth grating, as shown in Figure 8c. Figure 8c refers to CLPG_2_, it depicts the power change of the M_1_ mode, ΔP_M1ΔT_, along the six gratings of Table 6, globally optimized. The grating R_7_ is removed because its contribution is deleterious and only six gratings are enough for sensor optimization. The maximum power change ΔP_M1_ at the end of the six gratings CLPG_2_, for a temperature variation from 0 °C to 100 °C, is |ΔP_M1_| = 33.46 mW. The deleterious effect caused by adding the seventh grating is apparent in Figure 8d. It depicts the power change of the M_1_ mode, ΔP_M1ΔT_, along the cascade made of six gratings of Table 6 and the seventh grating of CLPG_1_.

In this case, the power change ΔP_M1_ at the end of the seven gratings, for a temperature variation from 0 °C to 100 °C, is |ΔP_M1_| = 4.58 mW. A further sensor optimization by choosing a suitable length LΔPM1max for $R_{7}$ is not possible.

**Figure 9:**
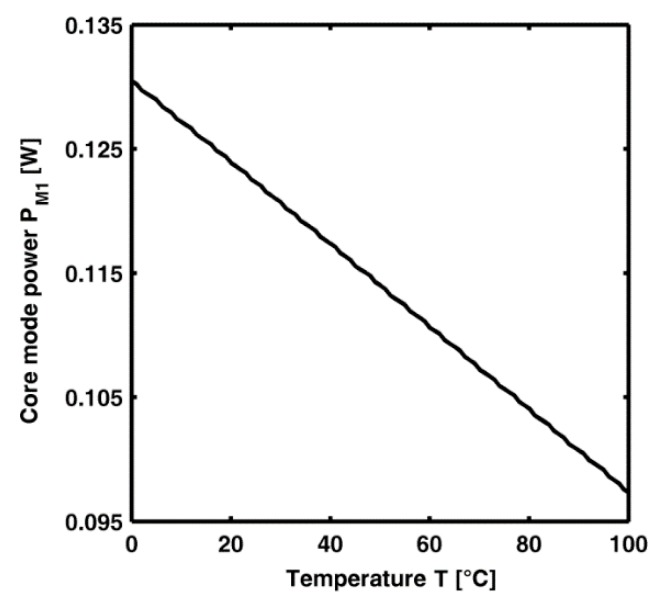
Power P_M1_ of the M_1_ mode at pump wavelength *versus* temperature at the output section of CLPG_2_, *P_p_* = 1 W.

Figure 9 illustrates the power P_M1_ of M_1_ mode *versus* the temperature at the output section of CLPG_2_ constituted by the cascade of the first six gratings listed in Table 6. It is worth noting that, after the proper CLPG_2_ design, a sensitivity S = |ΔP_M1_|/ΔT = 0.3346 mW/°C is obtained. Unfortunately, the Section A sensor cannot be directly used as temperature sensor because of the high zero offset. In fact, *S* = 0.3346 mW/°C is obtained around a core mode power mean value close to MV = 112 mW. The ratio S/MV is about 0.003 °C^−1^, it indicates a not feasible measurement condition by employing conventional/low cost optical power meters. Further sensor stages are thus required.

In the Section B sensor, the residual power of the cladding modes is completely attenuated. To obtain this behavior, this part of the sensor is optimized via the FEM electromagnetic investigation. In other words, the Section B sensor is designed to cut off the 32 cladding mode. The fiber geometry is defined to allow the single fundamental mode M_1_ propagation. In this way, further power exchanges between the cladding and the core is prevented. The two differences between Section A and Section B of the sensor are the following: (i) in Section B there are not the large external air holes and (ii) the pitch of the internal air hole of Section B is increased with respect to that of Section A. The cladding modes are cut in the Section B sensor MOF after a length of a few centimeters (corresponding to a high number of wavelengths). The cores of the Section A sensor and Section B sensor have the same optical and geometrical parameters. Therefore, there is a very good M_1_ mode matching when the light travels from the Section A sensor to the Section B sensor, *i.e.*, without reflections.

The Section C sensor is an ytterbium doped laser cavity, with a dopant concentration *N_Yb_* = 5 × 10^25^ ions/m^3^ [28]. The losses at the pump and at the signal wavelength are *γ*(*λ_P_*) = 4.2 dB/Km and *γ*(*λ_S_*) = 2.0 dB/Km, respectively. The main parameters, suitably optimized as in [29,30], are reported in Table 3. The optical cavity length is *L* = 3 m and the two FBGs operate as input and output mirrors with the reflectivity R_1_ = 0.99 and R_2_ = 0.06, respectively. The Section C sensor is optically pumped by M_1_ power at the wavelength *λ_P_* = 976 nm. The pump power at the end of CLPG_2_ depends on temperature. Then, it travels unperturbed through the Section B sensor. The output signal laser is obtained at the wavelength *λ_S_* = 1060 nm. By simulation, the threshold pump power is close to *P_th_* = 103.5mW. Figure 9 shows that the power at the output of Section B sensor, at the temperature T = 100 °C is *P_M1_* = 97.33 mW, which is lower than the threshold pump power. To obtain a fundamental mode power P_M1_, at the output of the Section B sensor, slightly larger than *P_th_*, the correct total power launched at the input of the Section A sensor, *P_P_* = 1.1 W, is calculated. In this case the maximum P_M1_ = 143.5 mW is obtained at T = 0 °C while the minimum *P_M1_* = 107.1 mW is obtained at T = 100 °C.

**Figure 10:**
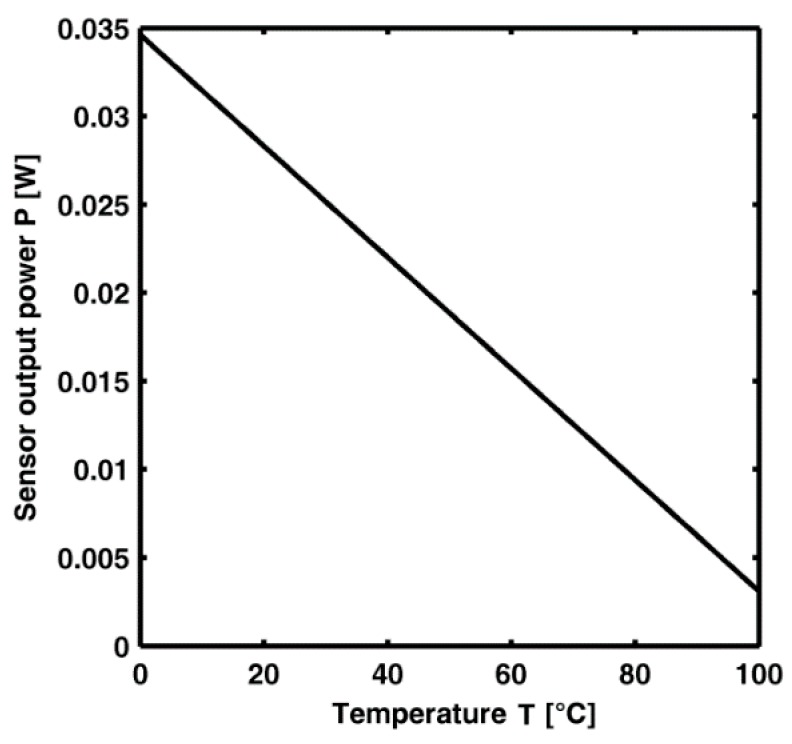
Sensor output *versus* the temperature for the pump power *P_p_* = 1.1 W.

The simulation of the sensor characteristic, *i.e.*, the output power *P* = *P_s_* at the signal wavelength *λ_S_* at the end of the Section C sensor *versus* the temperature, for an input *P_P_* = 1.1 W at the input of the Section A-sensor, is reported in Figure 10.

The output signal power is *P* = 34.60 mW at T = 0 °C and P = 3.087 mW at T = 100 °C. The linearity is apparent. The sensitivity S is very high:

$$S=\left|\frac{\Delta P}{\Delta T}\right|=315.1\;\mu W/C$$

The ratio S/MV is about 19.69 °C^−1^, it indicates a very good potential for temperature measurement by employing conventional optical power meters.

We underline that the design of: (i) the fiber section such as (ii) the number, the period and the lengths of the gratings of the cascade and (iii) the optimization of the operation condition, *i.e.*, the tuning of the pump in wavelength and power, allow us to finely handle/control the sensor response both during the design and its utilization. This aspect, in addition to the theoretically calculated high performance makes the proposed device particularly interesting. As an example, a version of this kind of sensor could be designed via the same procedure by employing different materials, e.g., radiation hardened glasses, for application in nuclear environments and for sensing different temperature ranges.

## 4. Conclusions

A novel MOF temperature sensor is conceived and accurately designed. The simulated sensitivity *S* = 315.1 μW/°C is good enough for actual applications. By considering the theoretical results obtained in this paper, for the complete measurement, in addition to the proposed sensor, only a low cost diode pump emitting at wavelength λ = 976 nm and a simple optical power meter are required. The proposed solution is very versatile. A version of this kind sensor could be designed via the same procedure by employing different materials and for sensing different temperature ranges.
